# Baseline glucocorticoids are drivers of body mass gain in a diving seabird

**DOI:** 10.1002/ece3.1999

**Published:** 2016-02-16

**Authors:** Holly L. Hennin, Alicia M. Wells‐Berlin, Oliver P. Love

**Affiliations:** ^1^ Department of Biological Sciences and Great Lakes Institute for Environmental Research (GLIER)University of WindsorWindsorOntarioCanada; ^2^ U.S. Geological SurveyPatuxent Wildlife Research CenterLaurelMaryland

**Keywords:** Baseline corticosterone, body mass, captive study, manipulation, white‐winged scoter

## Abstract

Life‐history trade‐offs are influenced by variation in individual state, with individuals in better condition often completing life‐history stages with greater success. Although resource accrual significantly impacts key life‐history decisions such as the timing of reproduction, little is known about the underlying mechanisms driving resource accumulation. Baseline corticosterone (CORT, the primary avian glucocorticoid) mediates daily and seasonal energetics, responds to changes in food availability, and has been linked to foraging behavior, making it a strong potential driver of individual variation in resource accrual and deposition. Working with a captive colony of white‐winged scoters (*Melanitta fusca deglandi*), we aimed to causally determine whether variation in baseline CORT drives individual body mass gains mediated through fattening rate (plasma triglycerides corrected for body mass). We implanted individuals with each of three treatment pellets to elevate CORT within a baseline range in a randomized order: control, low dose of CORT, high dose of CORT, then blood sampled and recorded body mass over a two‐week period to track changes in baseline CORT, body mass, and fattening rates. The high CORT treatment significantly elevated levels of plasma hormone for a short period of time within the biologically relevant, baseline range for this species, but importantly did not inhibit the function of the HPA (hypothalamic–pituitary–adrenal) axis. Furthermore, an elevation in baseline CORT resulted in a consistent increase in body mass throughout the trial period compared to controls. This is some of the first empirical evidence demonstrating that elevations of baseline CORT within a biologically relevant range have a causal, direct, and positive influence on changes in body mass.

## Introduction

Variation in individual state (e.g., body condition/energetic stores) is the source of numerous life‐history trade‐offs (Stearns 1992) and can act as both a constraint, and a driver, of fitness‐related life‐history decisions (McNamara and Houston 1996). During energetically demanding life‐history stages (i.e., migration, reproduction), individuals with greater energetic stores (i.e., higher body condition) are predicted to complete these stages with greater success (Stearns 1992; Rowe et al. 1994; McNamara and Houston 1996; Kisdi et al. 1998). For example, individuals with higher body mass are often able to arrive earlier on the breeding grounds and reproduce earlier and with greater success (Lepage et al. 2000; Bêty et al. 2003; Gladbach et al. 2010; Descamps et al. 2011). Although individual variation in the rate at which accumulating these energetic stores (i.e., lipids) is theorized to have a major impact on reproductive timing and investment (Bêty et al. 2003; McNamara and Houston 2008), there is scant empirical data on the underlying mechanisms driving the procurement and accumulation of endogenous resources (Hennin et al. 2015).

Baseline glucocorticoids (GCs – corticosterone/cortisol) are hormones found in all vertebrates that mediate variation in energetic demand (Dallman et al. 1993; Landys et al. 2006). As such, baseline GCs experience daily and seasonal variation, with higher levels being associated with more energetically demanding life‐history stages (Romero 2002; Landys et al. 2006; Crespi et al. 2013). Baseline GCs are elevated when resources are scarce or individuals are in a negative energetic state (Love et al. 2005; Kitaysky et al. 2007; Jenni‐Eiermann et al. 2008), presumably to stimulate foraging behavior and subsequent resource acquisition (Lõhmus et al. 2006; Crossin et al. 2012). In individuals preparing for energetically demanding life‐history stages, elevated baseline GCs within biologically relevant levels result in higher foraging rates (Astheimer et al. 1992; Kitaysky et al. 1999; Lõhmus et al. 2006; Angelier et al. 2007; Crossin et al. 2012), increased rates of condition gain (Crossin et al. 2012), and larger lipid stores (Holberton 1999; Holberton et al. 2007), all of which can positively influence fitness (Crossin et al. 2012). Nonetheless, it is important to remember the dual nature of elevated GCs (Lõhmus et al. 2006), especially when manipulating them in wild organisms (Crossin et al. 2015). When GCs are experimentally elevated outside the baseline range – such as during an acute stress response or *via* pharmacological manipulation, for example – they promote the *expenditure* of lipid stores to fuel survival‐related behaviors (Wingfield et al. 1998; Breuner and Hahn 2003), thereby resulting in a *negative* proximate impact on body condition (Criscuolo et al. 2005; Bourgeon and Raclot 2006; Jenni‐Eiermann et al. 2008; Angelier et al. 2009, 2010). As such, while lower, biologically relevant elevations of GCs in a baseline range are expected to have *positive* impacts on individual state (Crossin et al. 2015; Hennin et al. 2015), greater elevations are expected to induce an emergency life‐history stage or stress response, and have a short‐term *negative* impact on individual state (Wingfield et al. 1998).

We manipulated baseline corticosterone (CORT, the primary avian GC) in a captive colony of white‐winged scoters (*Melanitta fusca deglandi*), a northern breeding, diving seaduck. Our overall goal was to examine whether variation in baseline GCs acts as a causal mechanism for positive impacts on individual state in a species that gains lipid stores prior to migration and commencing breeding. Our specific aims were to (1) examine whether a biologically relevant exogenous increase of CORT within a baseline range resulted in positive changes in body mass (i.e., increases in body fat), (2) determine whether this effect was due to either positive effects of elevated baseline CORT (i.e., *via* predicted increases in resource acquisition) or the often presumed “inhibitory” effects of elevated baseline CORT (i.e., *via* negative feedback through the hypothalamic–pituitary–adrenal (HPA) axis), and (3) determine whether increases in body mass were reflected by an increase in plasma triglycerides (a measure of physiological fattening rate). For the latter, we focused on plasma triglycerides (TRIG) as they are a measure of the lipids synthesized by the liver used for depositing fat stores endogenously (Jenni and Schwilch 2001; Cerasale and Guglielmo 2006; Zajac et al. 2006; Anteau and Afton 2008) and when corrected for body mass, indicate an individual's physiological fattening rate (Williams et al. 2007).

## Materials and Methods

### Study site and colony

Work was conducted between January and April 2013 using a captive colony of adult white‐winged scoters (hereafter scoters; male: *n* = 5; female: *n* = 8) housed at the PWRC (Patuxent Wildlife Research Center) in Laurel, MD, USA. Birds were kept in mixed‐sex, outdoor pens covered with shade cloth. Pens were 11.5 m^2^ with gravel substrate and a conical pond (2.1 m diameter, ~1 m deep at center) with continual flowing freshwater. Experimental pens were separated from each other by at least two nonexperimental pens to reduce researcher influences on GCs and bird behavior. Birds were maintained on an *ad libitum* diet of Mazuri seaduck diet pellets (PMI Nutrition International) to ensure that no variation in body mass or fattening rates could be attributable to differences in diet composition (Seaman et al. 2005; Cerasale and Guglielmo 2006). All maintenance and experimental procedures were approved through both the PWRC (ACUC approval for “Corticosterone, energetics and individual state in diving seaducks”) and University of Windsor (AUPP #12‐15: “Mechanisms behind variation in individual state in diving seaducks) animal care and use committees.

### Experimental design, blood sampling, and corticosterone manipulation

Three separate 21‐day trials were performed. Individuals were assigned a randomized implantation schedule *via* random number generator across trials for each of our three treatment pellets (Innovative Research of America, Sarasota, FL) for a repeated‐measures design: control pellet (15 mg containing cholesterol), a low dose of CORT (“low CORT”; 15 mg pellet of CORT in a cholesterol matrix), and a high dose of CORT (“high CORT”; 35 mg pellet of CORT in a cholesterol matrix). The experimental manipulation was designed to elevate corticosterone levels within a biologically relevant baseline range (wild white‐winged scoters: 6.64 ± 1.19 ng mL^−1^; range 0.51–46.7 ng mL^−1^; Palm et al. 2013), and not to elevate levels to those seen during an acute stress response. Indeed, we found that the plasma levels of our manipulated birds fell well within this baseline range (range 1.00–33.4 ng mL^−1^; see Results). Although pellets are designed to last 21 days in mammals, based on previous studies in birds, we expected them to last approximately 14 days or less given their higher basal body temperature (see Bonier et al. 2007; Müller et al. 2009a,b). We therefore sampled individuals every 3 days for a total of 14 days (with the exception of day 5 of the experiment; see *ACTH Challenge* details). Birds were given a week of rest before initiating the next trial.

Blood sampling took place between 0800 h and 1200 h to control for any potential diel variation in baseline corticosterone. Individuals in a pen were sampled together within 3 min of researchers being in sight of the pens to obtain conservative baseline samples (Romero and Reed 2002) and then weighed (g). Blood samples were collected by puncturing the tarsal vein using a 26‐G needle and 75‐*μ*L heparinized capillary tubes. Blood samples were placed in heparinized storage tubes and centrifuged at 2000 g for 10 min. Plasma was separated from the red blood cells and stored separately at −80°C until further analysis. Tarsus measurements (mm) were taken on the first day of sampling.

On the first day of an experimental trial, birds were implanted with treatment pellets at the base of the thigh in an area of lose skin. The leg in which birds were implanted alternated after each trial. After sanitizing the implant area with Betadine, the implant site was anesthetized locally with 0.36 mg kg^−1^ dose of 5 mg mL^−1^ bupivacaine (Hopsira, Montréal, QC, Canada) using a 25‐G needle. After the local anesthetic had taken effect (approx. 5 min) and the area was re‐sterilized with Betadine, a small incision slightly larger than the pellet was made in the skin using a #15 scalpel blade, and a pocket in the subcutis was made for the pellet by gently separating the skin from the muscle. After the appropriate treatment pellet was inserted under the skin, the wound was closed using 2–3 sutures of UV degradable monofilament (PDS* II (polydioxanone) suture; Ethicon, Markham, ON, Canada). The surgical area was re‐sanitized, and the bird was released back into its pen. The surgical site was monitored throughout the trial to ensure that it was healing properly and that there was no infection. In seven instances (five individuals in trial 2 and 3 in trial 3), individuals rejected the implanted pellets, encapsulating and extruding the pellets. As a result, the dosage of CORT to the individual was ambiguous and unstandardized. Therefore, the data from those individuals within that trial were excluded.

### Adrenocorticotropic hormone (ACTH) challenge

On the fourth and fifth days of the experiment for each trial, birds underwent an ACTH hormonal challenge to test the responsiveness of the HPA axis (Noirault et al. 1999; Faure et al. 2003; Nilsson et al. 2008). Following a blood sample to assess baseline CORT, birds were injected with either 100 IU kg^−1^ of porcine ACTH (Sigma, St. Louis, MO, USA) dissolved in 0.5 mL of lactated Ringers solution (Sigma) (high CORT: *n* = 4, control CORT: *n* = 5), or 0.5 mL of lactated Ringers solution (hereafter referred to as “saline”) as a control (high CORT: *n* = 4, control CORT: *n* = 3) into the breast muscle using a 25‐G needle‐equipped syringe. Birds received either ACTH or saline solution on day 4 and the opposite injection on day 5 to also obtain individual variation in responses to the injections. Birds were then placed in individual carrier crates in a darkened, outdoor area, and blood was sampled 30 and 60 min postinjection before being released back into their pens. Blood samples were centrifuged at 10,000 rpm for 10 min, and the plasma and red blood cells were stored separately at −80°C until further analysis.

### Physiological assays

Baseline corticosterone (CORT) was measured using a commercially available, enzyme immunoassay kit based on competitive binding and previously validated in diving seaducks (EIA; Assay Designs, Ann Arbor, MI) (Hennin et al. 2015) and was optimized for white‐winged scoters (Palm et al. 2013). Samples were un‐extracted and run in triplicate at a 1:40 dilution with a 1.5% steroid displacement buffer. Each plate included a CORT‐spiked control sample and a standard curve produced by serially diluting a kit‐provided, 200,000 pg mL^−1^ CORT standard, and plates were read at 405 nM wavelength (for details see Hennin et al. 2015). The inter‐ and intra‐assay coefficient of variation across all plates was 2.66% and 7.78%, respectively.

Plasma triglycerides (TRIG) were measured using a commercially available kit (Sigma‐Aldrich, Oakville, ON, Canada) previously validated in diving seaducks (Hennin et al. 2015). We used a 1:2 dilution for samples before adding them to a 96‐well microplate in duplicate. Each plate included a serially diluted standard curve of glycerol standard (2.54 mmol L^−1^) and a control of laying hen plasma (Sigma‐Aldrich Canada, Oakville, ON, Canada). Reagent A was first added to measure free glycerol, followed by Reagent B to measure total glycerol. After the addition of each reagent, the plates were left to shake for 10 min at 37°C and then read using a plate reader at 540 nM wavelength. The amount of triglycerides (mmol L^−1^) was calculated by subtracting the amount of free glycerol found in the first plate read from the amount of total glycerol found in the second plate read. Inter‐ and intra‐assay coefficients of variation were 7.61% and 8.32% for total TRIG, and 7.27% and 4.68% for free glycerol, respectively. Final TRIG values were corrected for body mass to obtain residuals, indicating fattening rates (Williams et al. 2007).

### Statistical analyses

We tested for differences in baseline CORT and size‐corrected body mass between treatments and between sexes at the start of the overall experiment (beginning of trial 1) using an ANOVA (analysis of variance). To test for trial‐induced changes in baseline CORT and potential seasonal effects, we ran an ANOVA with trial number as an independent variable to test for differences across trials in baseline CORT. Due to lower than expected blood sample numbers, in this analysis, we were only able to include one of an individual's measures from each of the three trials (no repeated measures) while maintaining balanced sample sizes across trials. Data points used in the analyses were selected randomly using a random number generator. Data from all trials were included in the analysis as the influence of CORT pellets within a focal trial did not influence baseline CORT values in subsequent trials (see Results). We ran a general linear mixed‐effects model with individual as a random effect to test for trial‐induced changes in size‐corrected body mass at the start of each trial (comparing day 1 measures for all three trials). Size‐corrected body masses appeared to demonstrate a trend toward increasing in the second and third trials compared to the first trial (trial: *F*_2,8.48_ = 3.78, *P* < 0.06; trial 1: 1152.1 ± 42.8 g, *n* = 9, trial 2: 1213.0 ± 42.5 g, *n* = 8, trial 3: 1216.2 ± 44.0 g, *n* = 6). We therefore suspected that the washout period between trials was too short, potentially biasing the treatment's apparent impact on body mass in subsequent trials. As such, to be as conservative as possible, we excluded trials 2 and 3 from analyses involving body mass and fattening to prevent any potential biases.

**Figure 1:**
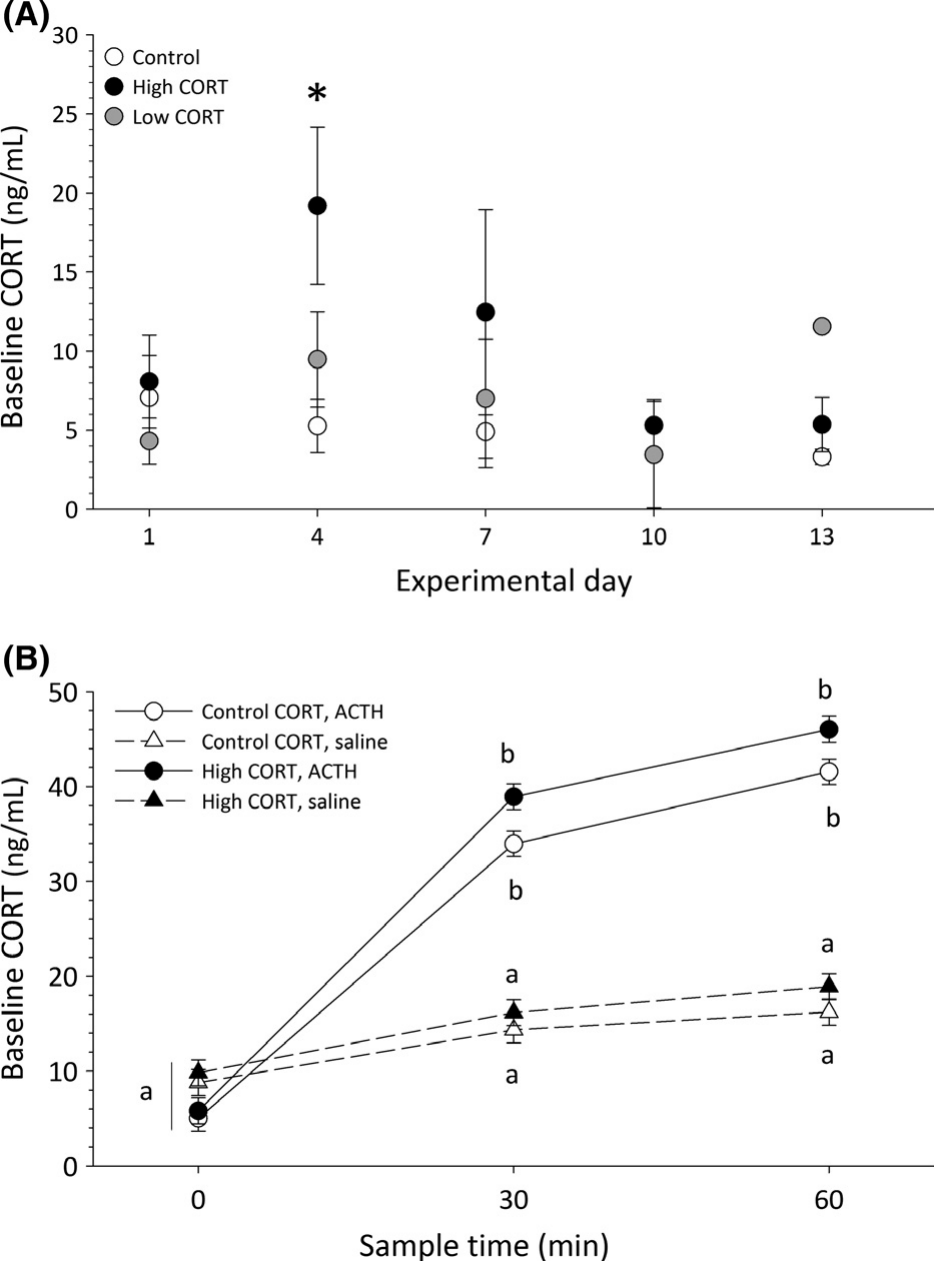
Influence of treatment (exogenous baseline CORT elevation vs. control) (A) and ACTH injection (B) on baseline levels of corticosterone in captive white‐winged scoters. Data included in analyses are presented as the model‐corrected least square means and standard errors. Values presented for CORT are back‐transformed from a log transformation. Asterisk indicates a significant difference between the high CORT group and control group only. Refer to Table S1 for sample sizes (A).

The peak concentration of plasma CORT due to the treatment pellets was predicted to be approximately 4 days postimplantation (Müller et al. 2009a). To test whether the low CORT and high CORT treatments elevated baseline CORT differentially compared to the control group, we ran two‐one‐tailed t‐tests comparing the control to each treatment group separately on day 4. The low CORT treatment did not significantly differ from the control group on day 4 (*t* = 0.73, df = 5.78, *P* < 0.25, control: *n* = 4; low CORT: *n* = 4; Fig. 1A), indicating it was unsuccessful in elevating CORT. Further, we found only a moderate effect size (Cohen's *d* = −0.52, *r* = −0.25). We therefore excluded the low CORT group from all subsequent analyses as we were aimed to test the influence of baseline elevations of CORT on changes in mass. We performed a general linear mixed‐model analysis to examine the responsiveness of the HPA axis to the ACTH challenge, with individual included as a random effect. We also included the fixed effects of treatment group, sampling time (initial baseline sample, 30/60 min postinjection), ACTH treatment, and an interaction between ACTH treatment and sampling time (with a Tukey's post hoc test). We found no effect of sex on differences in response to the ACTH trials (*F*_1,7.85_ *= *0.35, *P* = 0.57) and therefore pooled sexes for ACTH trial analysis.

The change in body mass was calculated as the difference between the body mass on a given experimental day and that at the start of the trial, making the comparison of the relative changes across individuals and sexes possible. Change in body mass was analyzed using a mixed‐effects generalized linear model including individual as a random effect, and as fixed effects CORT treatment group, experimental day, sex, and the interaction between CORT treatment and experimental day. We ran a Tukey's post hoc test to test for differences between groups in the interactive effect. Changes in physiological fattening rate (TRIG) were analyzed using a general linear model including experimental day and treatment group as independent variables. As it is likely that impacts of elevated baseline CORT on plasma TRIG are temporally delayed after foraging, we ran two models: one to compare day 1 one and 4 (expected day of peak CORT secretion) and a second to compare days 1 and 7 (next sampling day after peak secretion). We included only one of an individual's measures across trials to maintain balanced sample sizes across trials. Due to low sample sizes, we were unable to include sex as a variable in fattening rate analyses. Baseline CORT values were log‐transformed for normality in analyses and back‐transformed for figures and to report means and standard errors. All data met the assumptions for a parametric test. All analyses were run using JMP 10.0 (SAS Institute, Cary, CA). Results are presented as means ± SEM unless otherwise stated.

## Results

### Changes in baseline CORT and HPA axis activity

There were no differences between the three treatment groups (*P* > 0.77; Fig. 1A) or between sexes (*P* > 0.59) in their initial baseline CORT values at the start of the overall experiment (*F*_2,6_ = 0.23, *P* > 0.80, *n* = 9), and no differences in baseline CORT on the first day of each trial (*F*_2,9_ = 0.42, *P* > 0.66, *n* = 9; trial 1: 4.72 ± 1.44 ng mL^−1^, *n* = 6; trial 2: 2.90 ± 1.55 ng mL^−1^, *n* = 4; trial 3: 4.95 ± 1.87 ng mL^−1^, *n* = 2). However, baseline CORT was significantly elevated in the high CORT treatment group compared to control birds by day 4 of the experiment (*F*_2,7_ = 12.66, *P* < 0.005, *n* = 10 (control = 6, high CORT = 4); Fig. 1A; Table S1), with a relatively large effect size (Cohen's *d* = −2.14, *r* = −0.73). Further, males had higher baseline CORT levels than females (males: 13.48 ± 1.28 ng mL^−1^, *n* = 5; females: 4.82 ± 1.31ng mL^−1^, *n* = 5; *P* < 0.03). As predicted, individuals administered ACTH had significantly higher CORT levels 30 and 60 min postinjection compared with saline injections (sampling time‐ACTH treatment interaction: Table 1, 2; Fig. 1B). However, both treatment groups responded to the ACTH challenge similarly (Table 1; Fig. 1B), indicating that the exogenous CORT treatment had no significant effect on the responsiveness of the HPA axis.

**Table 1:** Summary of fixed effects for ACTH trials, change in body mass, and fattening rate (plasma TRIG) analyses in response to an exogenous elevation of baseline corticosterone in captive white‐winged scoters. Bolded values indicate significant effects

Analysis	Variable	*F*	df	*P*
ACTH Trial	CORT Treatment	0.3	1, 40.58	0.59
	ACTH Treatment	4.84	1, 38.41	**0.03**
	Sample Time	28.7	2, 30.83	**0.0001**
	Sample Time*ACTH Treatment	8.88	2, 30.83	**0.0009**
Change in body mass	Experiment Day	8.68	4, 21.35	**0.0003**
	Treatment	60.17	1, 3.43	**0.0003**
	Sex	1.15	1, 3.42	0.35
	Experiment Day*Treatment	4.02	4, 21.35	**0.01**
Fattening rate 1–4	Experiment Day	2.69	1	0.18
	Treatment	0.21	1	0.67
Fattening rate 1–7	Experiment Day	1.11	1	0.37
	Treatment	3.89	1	0.14

**Table 2:** Parameter estimates for ACTH trial, change in body mass, and fattening rates (mass‐corrected plasma TRIG) between experimental day 1 to day 4 and day 1 to day 7 for white‐winged scoters. Bolded values indicate significant effects

Analysis	Parameter	Estimate	SE	df	*t*	*P*
ACTH Trial	Intercept	1.22	0.08	7.21	15.99	**0.0001**
	CORT Treatment (Control)	−0.03	0.05	40.58	−0.55	0.59
	ACTH Treatment (ACTH)	0.09	0.04	38.41	2.2	**0.03**
	Sample Time (T0)	−0.37	0.05	30.83	−7.53	**0.0001**
	Sample Time (T30)	0.15	0.05	30.83	3.04	**0.005**
	Sample Time (T0)*ACTH Treatment (ACTH)	−0.21	0.05	30.83	−4.21	**0.0002**
	Sample Time (T30)*ACTH Treatment (ACTH)	0.1	0.05	30.83	2.01	**0.05**
Change in body mass	Intercept	32.15	3.75	3.35	8.56	**0.002**
	Experiment Day 1	−31.10	7.18	21.21	−4.33	**0.0003**
	Experiment Day 4	−12.80	7.18	21.21	−1.78	0.09
	Experiment Day 7	−1.41	7.49	21.93	−0.19	0.85
	Experiment Day 10	16.84	7.18	21.21	2.34	**0.03**
	Treatment (Control)	−28.43	3.67	3.43	−7.76	**0.003**
	Sex (Female)	−3.93	3.67	3.43	−1.07	0.35
	Experiment Day 1*Treatment (Control)	28.17	7.18	21/21	3.92	**0.0008**
	Experiment Day 4*Treatment (Control)	−1.53	7.18	21.21	−0.21	0.83
	Experiment Day 7*Treatment (Control)	−9.48	7.49	21.93	−1.27	0.22
	Experiment Day 10*Treatment (Control)	−6.56	7.18	21.21	−0.91	0.37
Fattening Day 1–4	Intercept	−0.45	0.12	NA	−3.83	**0.02**
	Experiment Day 1	−0.19	0.12	NA	−1.64	0.18
	Treatment (Control)	0.05	0.12	NA	0.46	0.67
Fattening Day 1–7	Intercept	−0.15	0.16	NA	−0.92	0.43
	Experiment Day 1	−0.18	0.17	NA	−1.05	0.37
	Treatment (Control)	−0.34	0.17	NA	−1.97	0.14

### Change in body mass and fattening rate

**Figure 2:**
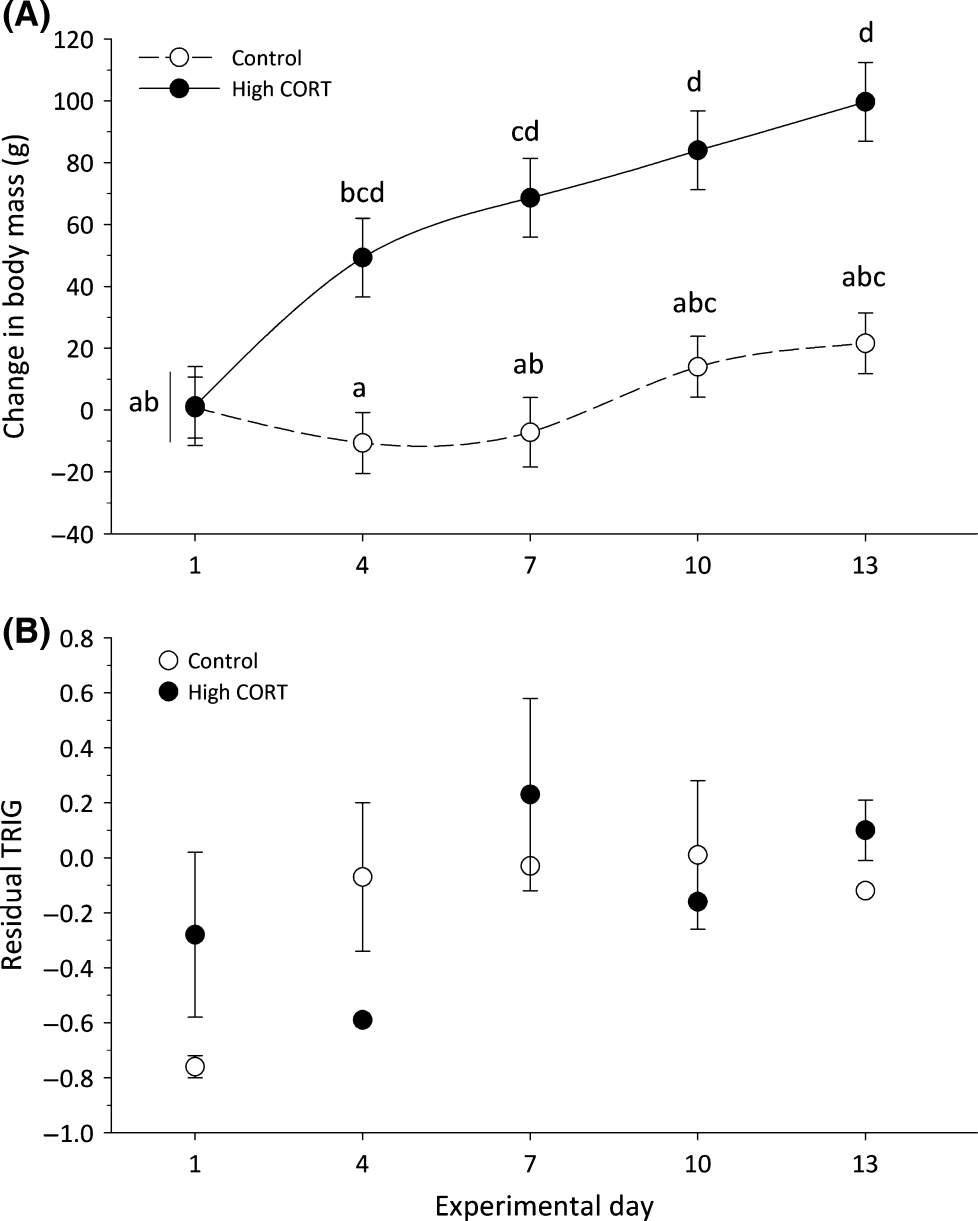
The impact of an exogenous elevation in baseline CORT on changes in body mass (A) and fattening rate (B) in captive white‐winged scoters across experimental days. Data included in analyses are presented as the model‐corrected least square means and standard errors. Letters indicate significant differences between groups. Refer to Table S1 for sample sizes (B).

We found no difference in size‐corrected body mass between experimental groups at the beginning of the first trial (*t* = 0.10, df = 6.81, *P* = 0.93, *n* = 11; control: 0.15 ± 1.18, *n* = 6; treatment: 0.37 ± 1.92, *n* = 5). However, we detected a significant two‐way interaction between treatment and experimental day on the change in body mass where individuals in the high CORT (*n* = 5) treatment had larger positive changes in body mass compared to control (*n* = 5) individuals (Tables 1, 2; Fig. 2A). Surprisingly, we did not detect a treatment effect on physiological fattening rates (TRIG corrected for body mass) between control and high CORT groups when comparing either day 1 to day 4 (control: *n* = 3; high CORT: *n* = 4; Tables 1, 2, S1; Fig. 2B) or day 1 to day 7 (control: *n* = 3; high CORT: *n* = 3; Tables 1, 2, S1; Fig. 2B).

## Discussion

We successfully experimentally elevated baseline corticosterone (CORT) in captive white‐winged scoters within a biologically relevant, baseline range without inhibiting the activity of the HPA axis. Although we only produced an ephemeral increase in baseline CORT, peaking on experimental day 4 with levels appearing to return to baseline levels between day 7 and day 10, individuals demonstrated a consistent and continual increase in body mass over the two‐week period of implantation. As such, we have discovered a direct and positive causal relationship between elevated baseline CORT and increases in body mass in a diving seaduck. Importantly, this baseline CORT‐mediated increase in body mass was due to a positive effect of elevated baseline CORT (i.e., likely *via* predicted increases in resource acquisition) and not an “inhibitory” effect of elevated exogenous baseline CORT resulting in negative feedback of the HPA axis. Nonetheless, this treatment‐related change in body mass was not mirrored by an increase in the physiological fattening rate.

### Corticosterone secretion dynamics

Only the high CORT treatment pellets significantly elevated CORT in our captive white‐winged scoters, although these levels were well within the expected natural baseline range for this species in the wild (6.64 ± 1.19 ng mL^−1^; range 0.51–46.7 ng mL^−1^) (Palm et al. 2013), indicating that our detected levels were within a biologically relevant, baseline range. We found that male white‐winged scoters had higher baseline plasma CORT than females at the peak of CORT release from the pellet. Pellets implanted in individuals were consistent in size and thus slightly variable across individuals. It is likely that males, which are larger on average than females, had a slightly higher metabolic rate than females, given the inherent positive relationship between body mass and metabolic rate (e.g., Nagy 2005). As such, if males had a higher metabolic rate, and individuals were administered an un‐scaled dosage of CORT, males may have metabolized the pellets more quickly, thereby exposing themselves to greater concentrations of CORT from the pellets than females. Although the pellets are manufactured to elevate CORT over a period of 21 days in mammals, we found a peak in CORT secretion 3 days postimplantation (i.e., on experimental day 4) with a rapid tapering off of CORT beginning approximately one week postimplantation. Studies in other avian species using this manipulation technique have found similar secretion trends in which there is a peak in plasma CORT concentrations shortly after implantation (1–3 days; Müller et al. 2009a) which slowly tapers off until returning to baseline levels (within approximately 7 days postimplantation; Bourgeon and Raclot 2006; Bonier et al. 2007; Almasi et al. 2008; Müller et al. 2009b).

There are three potential reasons for the rapid tapering off of CORT in these secretion profiles. First, it is possible that individuals were preventing the release of CORT either through encapsulation of the pellet or through clearing CORT from the circulatory system. We monitored the implantation site throughout each experimental trial to ensure the site was healing and for the presence of the pellets. Any individuals that had encapsulated pellets during the trial were removed from analyses due to the ambiguity in the nature of CORT secretion from the pellets while encapsulated. Second, through negative feedback individuals could have begun down‐regulating the endogenous production of CORT due to the rapid influx of exogenous CORT (Goutte et al. 2011). To test for this impact, we intentionally timed our ACTH trials to closely match the expected maximal secretion of CORT from the pellets to examine whether the HPA axis was still active and fully responsive. If the exogenous CORT was inhibiting the HPA axis, then the high CORT birds should have exhibited a significant depression in endogenous CORT secretion in response to the ACTH challenge. However, high CORT birds challenged with ACTH showed CORT responses that were not significantly different from those of ACTH‐injected control birds. This indicates that the HPA axis of high CORT birds was still active and able to secrete endogenous CORT normally. The final and most likely explanation for the relatively ephemeral nature of the CORT pellets is that they are primarily designed for use in mammals, and therefore, pellets were likely metabolized more quickly in avian species which exhibit higher relative metabolic rates (Müller et al. 2009a). Again, this assumption is supported by recent work using these pellets in a number of avian species (Bourgeon and Raclot 2006; Bonier et al. 2007; Almasi et al. 2008; Müller et al. 2009b).

### Linking elevated baseline corticosterone to changes in body mass

We found that individuals with exogenously elevated baseline CORT increased in body mass throughout the trial period. Although a number of studies have examined relationships between elevated baseline CORT and foraging behaviors (Astheimer et al. 1992; Dallman et al. 1993; Breuner et al. 1998), here we confirm that this CORT‐mediated effect on body mass was due to a direct *positive* effect of elevated baseline CORT, presumably *via* the previously observed increase in resource acquisition. It is key to note that we witnessed changes in mass in the high CORT birds, particularly later in the trial period, and no changes occurred in our control birds across the trial, indicating that these results were not due to seasonal influences but rather due to the treatment. Importantly, CORT‐mediated increases in body mass did not occur as a result of the elevated exogenous baseline CORT inhibiting the HPA axis, thereby reducing endogenous production of CORT with an associated downstream positive impact on body mass. Rather, individuals responded directly to the elevation of baseline CORT itself, with subsequent increases in body mass. It is well known that relationships between elevated baseline CORT and changes in body mass depend heavily on the interactions between life‐history stage, life‐history strategy, and the dosage of CORT administered in a given species (Crossin et al. 2015). For instance, yellow‐rumped warblers (*Setophaga coronata*) exposed to longer day lengths (i.e., simulating spring migration) exhibited elevated baseline CORT secretion with a temporally paired increase in body mass (Holberton 1999). Similarly, an experimental reduction in baseline CORT levels in dark‐eyed juncos (*Junco hyemalis*) resulted in less body mass gain than individuals with higher, normal baseline levels (Holberton et al. 2007). Finally, in wild macaroni penguins (*Eudyptes chrysolophus*), individuals with experimentally elevated baseline CORT exhibited increased foraging behavior and higher body mass gain (Crossin et al. 2012). Conversely, incubating common eiders (*Somateria mollissima*) with experimentally elevated CORT outside the normal baseline range (i.e., into the range of an acute stress response) lost significant amounts of body mass (Bourgeon and Raclot 2006), underscoring the dual nature of CORT secretion (Landys et al. 2006) and that the direction of this relationship is highly context dependent (Crossin et al. 2015). Although our results indicate that CORT acts as a “direct” mechanism driving an increase in body mass, CORT can also influence or be influenced by the secretion of other hormones (e.g., ghrelin, thyroid hormone, insulin) or neuropeptides (e.g., neuropeptide Y) which can have downstream consequences for foraging (Landys et al. 2006; Cornelius et al. 2013). As such, CORT may be a component of a complex set of mechanisms which can influence body mass, the strength of which may be dependent on life‐history stage, individual condition, and environmental signals (Landys et al. 2006; Cornelius et al. 2013).

Previous research examining the relationship between plasma TRIG and mass gain has found that elevated TRIG positively indicates an individual's body mass gain (“physiological fattening rate”: Jenni and Schwilch 2001; Cerasale and Guglielmo 2006; Anteau and Afton 2008; but see Dietz et al. 2009). We therefore predicted that if individual changes in body mass occurred in direct response to exogenous baseline CORT elevation, we would have also detected a corresponding increase in plasma TRIG. Interestingly, we detected no difference in plasma TRIG secretion between our control and high CORT groups on day 4 or day 7 postimplantation, nor were there any apparent trends in the data. Similar nontrends have been detected in red knots (*Calidris canutus*) (Dietz et al. 2009) and western sandpipers (*Calidris mauri*) (Seaman et al. 2005); however, in the latter case, this may have been due to methodological differences used to stimulate foraging and, therefore, changes in body mass (Seaman et al. 2005). Although we did not fast individuals to stimulate body mass gain, this lack of a trend may result from the time lag between foraging, digestion, and circulating triglycerides, with the concentration of plasma TRIG increasing throughout the day (Jenni and Jenni‐Eiermann 1996). Therefore, we may not have been able to detect a difference in our birds as they were sampled prior to feeding, exhibiting no relationship to treatment group despite the increased signal of energetic demand (i.e., elevated baseline CORT). Indeed, treatment differences in fattening rates may have been more detectible postfeeding in the early afternoon or evident through differences in foraging rates or amount of food consumed. Future studies seeking to establish causal links between baseline GCs, fattening rates and changes in body mass should ideally account for changes in foraging behavior if possible.

## Conflict of Interest

None declared.

## Supporting information


**Table S1**. Summary of sample sizes collected for corticosterone and mass‐corrected triglycerides (fattening rate).
