# Supplementary material for: Baseline glucocorticoids are drivers of body mass gain in a diving seabird
Source: Ecol Evol. 2016 Feb 16;6(6):1702–11. doi: 10.1002/ece3.1999 (PMC4755010; doi:10.1002/ece3.1999)
Supplement: Supplementary file 1 — Table S1. Summary of sample sizes collected for corticosterone and mass‐corrected triglycerides (fattening rate). [file ECE3-6-1702-s001.docx]

**Table S1**. Summary of sample sizes collected for corticosterone and mass-corrected triglycerides (fattening rate). All three trials of data were use for corticosterone analyses and presentation. Only the first trial was included in analyses and presentation for fattening rate (see Methods).

|  | **Corticosterone** | | |  | **Fattening Rate** | |  |
| --- | --- | --- | --- | --- | --- | --- | --- |
| **Experimental day** | **Control** | **Low CORT** | **High CORT** |  | **Control** | **High CORT** | |
| 1 | 5 | 6 | 3 |  | 3 | 3 | |
| 4 | 6 | 7 | 4 |  | 2 | 1 | |
| 7 | 4 | 4 | 3 |  | 1 | 2 | |
| 10 | 1 | 3 | 4 |  | 3 | 1 | |
| 13 | 4 | 1 | 4 |  | 1 | 2 | |
